# Investigation into the Short-Term Effects of Intra-Articular Allogenic PRP in the Equine LPS Model of Joint Inflammation: A Randomised Control Trial

**DOI:** 10.3390/ani16162477

**Published:** 2026-08-10

**Authors:** Michael J. S. Duggan, Clodagh Kearney, Andrea del Rincon, Margot C. Labberté, Milda Baltrimaite, Rory Gibney, Pieter A. J. Brama

**Affiliations:** 1 School of Veterinary Medicine, UCD Veterinary Hospital, University College Dublin, Belfield, D04 W6F6 Dublin, Ireland; 2 Trinity Centre for Bioengineering, Trinity Biomedical Sciences Institute, Trinity College Dublin, D02 R590 Dublin, Ireland

**Keywords:** equine, PRP, lipopolysaccharide, synovitis, joint, LPS, platelet-rich plasma, osteoarthritis

## Abstract

This study aimed to investigate the effects of allogenic platelet-rich plasma (PRP) injection in lipopolysaccharide (LPS) inflamed equine radiocarpal joints. A preliminary study using a 0.0625 ng LPS dose was performed in the middle carpal joint and compared within animal to a saline control to confirm that synovitis was consistently produced using this dose. The main PRP effect study involved a bilateral 0.0625 ng LPS inflammation model with a within-animal comparison of LPS plus PRP versus a LPS plus saline control. Serial synovial fluid analysis was performed on samples obtained at post-injection hours (PIH) 0, 8, 24, 72, and 168. Synovial fluid analysis and clinical parameters were further complimented by objective gait and thermographic analyses. The 0.0625 ng dose induced a statistically significant increase in the synovial TP and WBC concentrations from baseline (*p* < 0.001) and control (*p* < 0.001, *p* = 0.013) values at PIH 8 in the preliminary study. No significant reduction in clinical or inflammatory markers was found in the PRP effect study, but a statistically significant increase in TP (*p* < 0.001) and PGE_2_ (*p* < 0.001) from baseline and control at PIH 8 in the PRP group (*p* < 0.001) was identified, which returned to baseline by 72 and 24 h, respectively. The protocol used in this study generated PRP with a PDGF-BB concentration of 7889.76 pg/mL, TGF-ß1 concentration of 27,369.96 pg/mL and platelet count of 954 × 10^9^ cells/L. In conclusion, there does not appear to be any measurable anti-inflammatory effect of PRP treatment at this dose and treatment timing in this model of joint inflammation during the early inflammatory phase. Platelet-rich plasma appears to induce an increase in PGE2, although this increase was transient and self-limiting.

## Simple Summary

The aim of this study was to investigate the effects of allogenic platelet-rich plasma (PRP) injection in lipopolysaccharide (LPS) inflamed equine radiocarpal joints. The study incorporated a preliminary LPS feasibility study using a 0.0625 ng dose. A sample of joint fluid was obtained at five timepoints to enable the analysis. The results of the preliminary study show that the injection of a 0.0625 ng dose of LPS induced a measurable degree of inflammation. The main study found that PRP did not exhibit any anti-inflammatory effects as measured by the methods used in this model of joint inflammation.

## 1. Introduction

Osteoarthritis (OA) is a significant chronic disease process affecting millions of individuals, both humans and animals, worldwide [1,2,3], within which synovitis has been identified as both a risk factor for and a consequence of OA [4,5] and presents an early therapeutic intervention target. In humans, 30–50% of adults over 65 years old experience clinical signs of OA [6], and this is reflected in the equine industry where 60% of lameness is estimated to result from OA [7]. The widespread impact of OA highlights the importance of developing effective treatment options for both humans and animals, and recently, interest in orthobiologics such as platelet-rich plasma (PRP) has grown.

Platelet-rich plasma is a product derived from whole blood (WB) in which the red blood cells (RBCs) are removed and the platelet fraction concentrated above that of baseline blood values [8,9]. Platelets contain three types of granules (alpha (α), dense and lysosomal) each containing high concentrations of bioactive substances including growth factors and chemokines responsible for haemostasis, immune functions, regeneration, and inflammation [10].

The use of PRP in veterinary and human practice has increased exponentially in recent years and it is now commonplace in the treatment of equine orthopaedic and soft-tissue injuries [11,12,13,14]. It has also been used in treating persistent equine endometritis [15] and for the treatment of wounds [16,17]. However, the mechanism of action is poorly understood and studies have reported variable results with low-quality evidence in both humans and equids [11,18,19,20].

There are a vast array of described protocols for producing PRP using commercial and non-commercial techniques, which result in PRP products of variable composition and consistency, making comparison and standardisation difficult [13,21,22]. Allogenic freeze-dried PRP has been shown to be safe and similar to autologous preparations when injected into healthy equine joints [23]. The use of an allogenic preparation offers the significant advantage of a standardised final PRP product prepared using an optimal donor, which is particularly important given that intrinsic donor variables can affect the overall PRP quality [24].

The lipopolysaccharide (LPS) model has been used for many years to induce lameness in the horse [25] and is now a well-established synovitis model for the study of inflammatory joint disease and associated therapeutics [26,27,28,29,30,31,32,33]. The LPS model not only induces synovitis but also rapidly activates Toll-like Receptor 4 and CD14 expression initiating the innate immune response that contributes to the inflammatory processes associated with osteoarthritis [34,35,36]. However, LPS dosages vary widely, with doses greater than 0.5 ng resulting in pyrexia, depression, and non-weight bearing lameness, while doses less than 0.5 ng had less pronounced lameness and absence of systemic signs [26]. In a previous study, we demonstrated that an intra-articular dose of 0.125 ng LPS induced a reproducible inflammatory response while minimising systemic effects, suggesting that an even lower dose may be feasible [37]. It is generally accepted that inflammation plays a pivotal role in the development of OA, and modulation of the inflammatory response has become an important therapeutic target [38,39,40]. Platelet-rich preparations have demonstrated pro-inflammatory, anti-inflammatory and anabolic properties in LPS-stimulated synovial membrane explants [41,42] and pro-inflammatory effects on cartilage explants [43]. Since little progress has been made in the development of regenerative cartilage treatments, several newer treatments focus on the reduction in inflammation to counter the development of osteoarthritis while avoiding the negative side effects current anti-inflammatory treatments have.

The main objective of this study is to investigate the safety and efficacy of allogenic PRP in reducing joint inflammation in the horse using a 0.0625 ng LPS dose. We hypothesised that allogenic PRP would be safe and effective in reducing clinical and synovial markers of inflammation in an animal-controlled low-dose LPS model of joint inflammation.

## 2. Materials and Methods

### 2.1. Experimental Animals

Nine Thoroughbred racehorses (18 joints) were selected for inclusion in this randomised controlled study. Ages ranged from 6 to 18 years old (mean ± SD age 13 ± 4 years) with body weights ranging from 475 to 604 kg (mean ± SD weight 524 ± 44 kg). Eight mares and one gelding were included. Each horse was examined by 1 ECVS boarded large animal surgeon, one ECVS large animal surgery resident, and one veterinary intern; their carpal joints were deemed to be within normal limits both clinically and radiographically. Four standard radiographic projections were acquired (lateromedial, dorsopalmar, dorsolateral–palmaromedial oblique and dorsomedial–palmarolateral oblique). During experimental sampling the horses were kept in stables (4 m × 4 m) on wood shavings. Hay and water were provided ad libitum, and concentrates were fed once daily. During the “off sampling” periods, the horses had access to pasture as a herd and were stabled again the day prior to sampling. The day prior to LPS induction, the horses’ carpal regions were clipped bilaterally, and a reference mark was made at the level of the accessory carpal bone (ACB) using Tippex (Société BIC, Clichy, France); this mark was maintained for the duration of the study. The Velcro (Velcro Ltd., Sandbach, UK) pads for the EquiGait sensors were applied using double-sided tape and their position marked using clippers.

### 2.2. Study Design

The study design was approved by the animal research ethics committee of the University College Dublin and performed under an Irish Health Products Regulatory Authority (HPRA) licence (AE18982-P146) at University College Dublin’s Lyons Research Farm.

Since the literature reports a large variation in LPS dosage and effect, and partly attributes this to individual animal variation, a preliminary study was performed on the experimental cohort of horses selected for this study to ensure the lowest possible LPS dose to provide a reliable and adequate inflammatory response while optimising the welfare impact. This used a unilateral design with the injection of 0.0625 ng LPS into one randomly assigned middle carpal joint and 0.9% sodium chloride control in the contralateral middle carpal joint. This allowed objective evaluation of lameness and comparison of clinical parameters and basic synovial fluid parameters (TP, WBC) in a within-animal comparison of the selected LPS dose versus a saline control.

**Figure 1:**
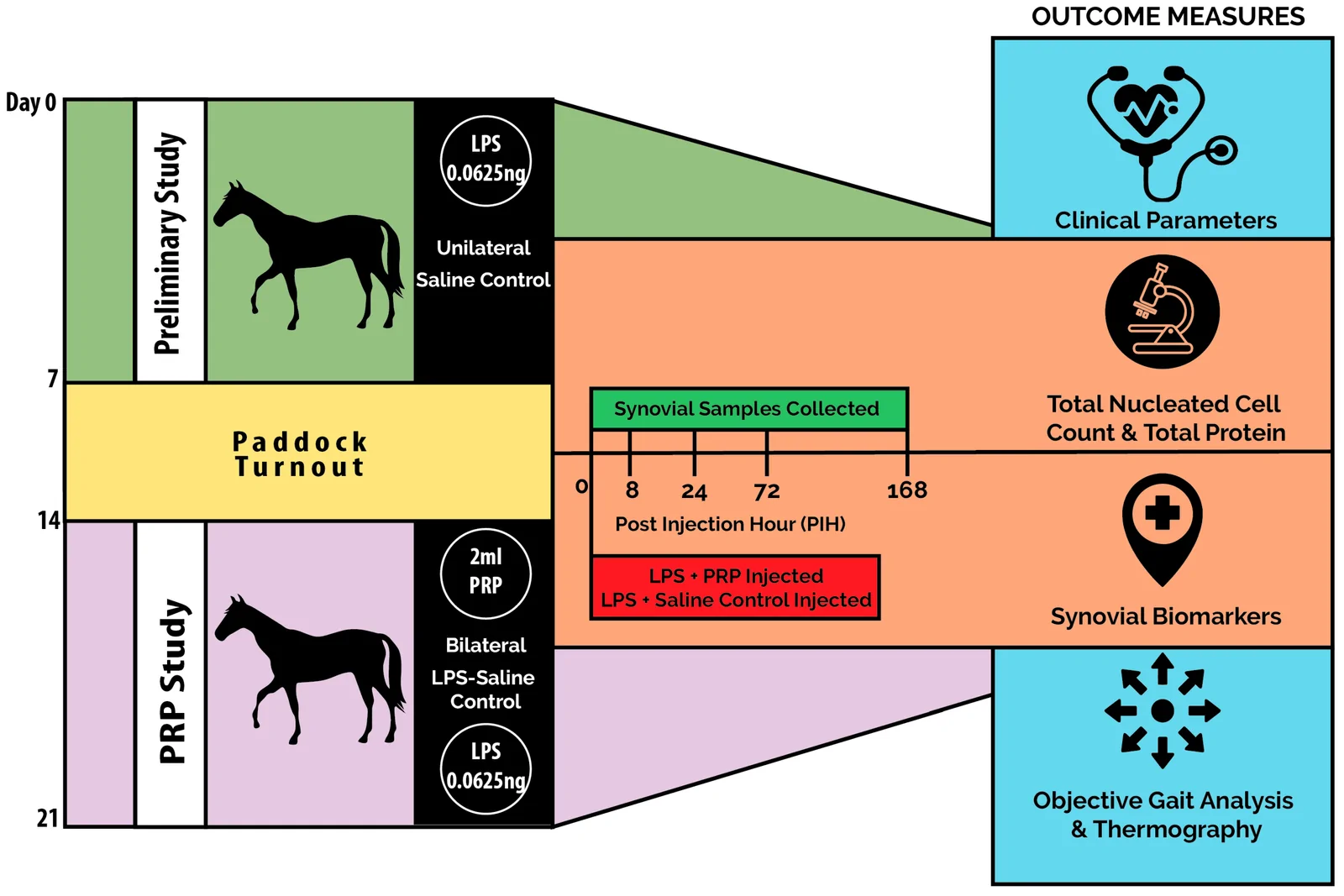
Schematic representation of the study design showing the preliminary study followed by the main PRP effect study. Synovial biomarker analysis was only performed in the main study.

The main part of the study was developed to investigate the efficacy of PRP treatment in reducing inflammation and the safety of PRP in inflamed joints. A bilateral model was used and involved inducing bilateral synovitis in the radiocarpal joints with the 0.0625 ng LPS dose. The joints were simultaneously treated with either 2 mL of PRP (as is a typical volume used clinically) or a 0.9% sodium chloride control. The bilateral within-animal controlled model was utilised to control for inter-animal variation, enhancing the experimental design and power to align with the 3 Rs, thus reducing animal usage. Clinical parameters were measured in addition to synovial cytological parameters and biomarkers, which were investigated using synovial fluid samples obtained at post-injection hours (PIH) 0, 8, 24, 72 and 168. The study design is illustrated in Figure 1.

The horses used in this study had previously been used to study the effects of two higher doses of LPS (0.25 ng/2.5 EU and 0.125 ng/1.25 EU) in the middle carpal joints. However, due to the authors’ concern of a potential overwhelming non-clinically relevant inflammatory response that could hinder the therapeutic potential of PRP, a lower dose was chosen to investigate in the preliminary phase of the present study. As per earlier findings in our research group [44], a period of 14 days between LPS inductions was allowed, which included a 7-day “washout” period where the horses were turned out to pasture without any interventions prior to commencing this study. This has been shown to allow synovial parameters to return to normal, baseline values. The radiocarpal joints of these horses used for the bilateral PRP study had not been injected with LPS previously.

### 2.3. Experimental Protocol

#### 2.3.1. Preparation of LPS

The LPS from Escherichia coli O55:B5 (L5418; Sigma-Aldrich Ireland Ltd., Arklow, Co. Wicklow Ireland; Lot: 045M4029V; Potency: 3,000,000 EU/mL) stock solution (3000 EU/mL) was prepared as previously described [37].

#### 2.3.2. Preparation of Platelet-Rich Plasma

**Figure 2:**
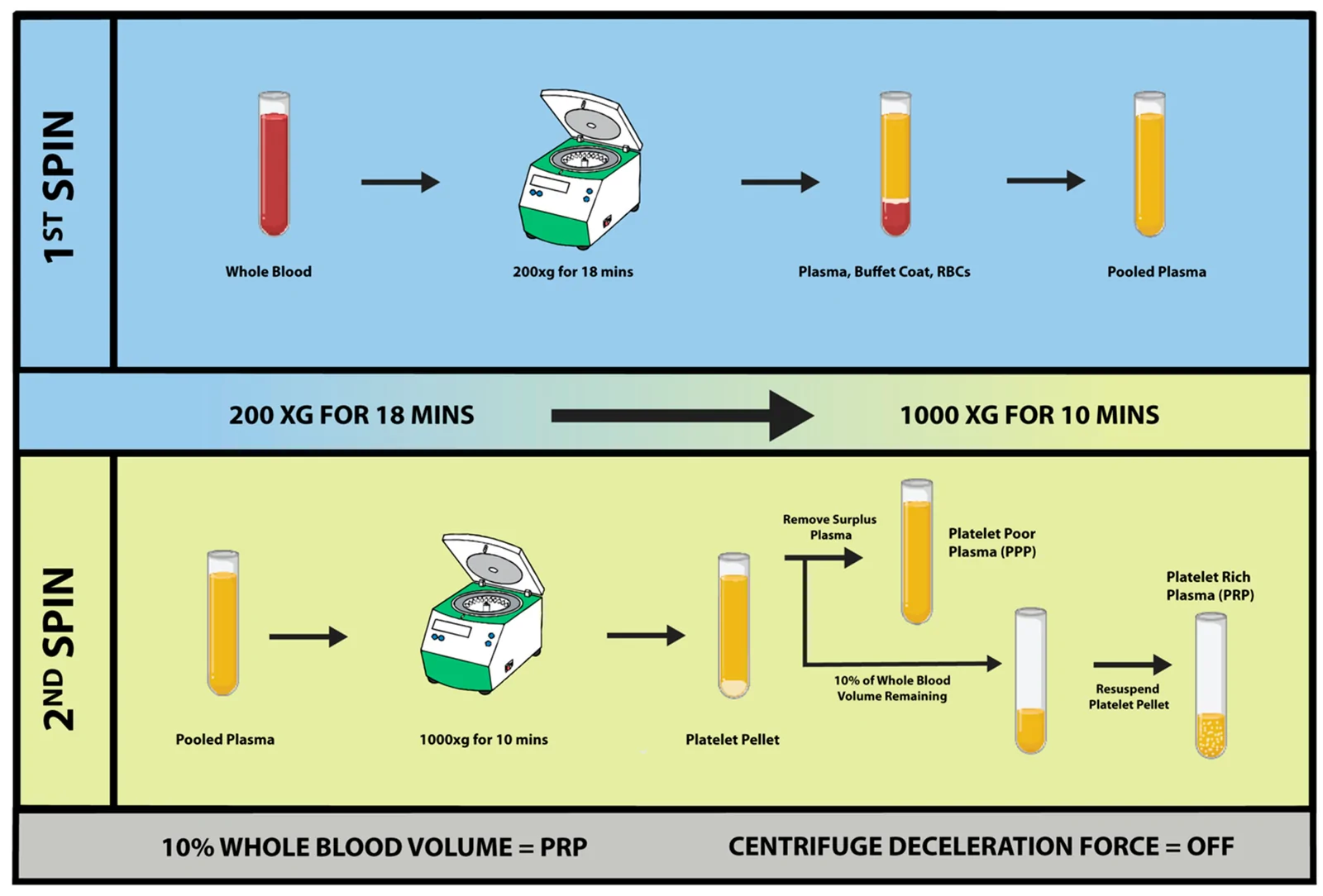
Schematic representation of the PRP production protocol outlining the key elements, including the 1st (200× *g* for 18 min) and 2nd (1000× *g* for 10 min) spin particulars.

Allogenic PRP was produced using a protocol adapted from Garbin et al. (2022) [23] [Figure 2]. The donor horse used was a 4-year-old Thoroughbred mare considered to be in good health and body condition. A 12-gauge intravenous cannula (Vygon) was placed in the left jugular vein and 450 mL of blood was collected into a blood collection bag containing CPD-A anticoagulant (Macopharma, Tourcoing, France). The bag was centrifuged at 200× *g* for 18 min. The supernatant was transferred from bag 1 to bag 3, avoiding disruption of the buffy coat and red blood cells. The supernatant was aliquoted into 50 mL conical tubes. The conical tubes were centrifuged at 1000× *g* for 10 min. Deceleration forces were off for all centrifugation steps. Excess supernatant was then removed to leave the remaining PRP (estimated to be 10% of the initial whole blood volume [17]), i.e., 50 mL whole blood gives 5 mL of PRP. The platelet pellet was resuspended within the remaining plasma. The PRP was aliquoted into 5 mL Luer lock syringes with sterile bungs applied, with 1 mL of air aspirated into the syringes before the bungs were applied to allow expansion when frozen. The PRP was frozen at −80 °C. Platelet-rich plasma analysis was performed using an automated analyser (Advia 2120, Siemens Healthcare Diagnostics Inc. New York, NY, USA), as previously reported by O’Shea et al., to be suitable for quantification of equine PRP platelet concentrations [45].

#### 2.3.3. Induction of Inflammation

In the preliminary phase of the study, the middle carpal joint was inflamed using a 0.0625 ng dose of LPS after collection of the PIH 0 synovial fluid sample as previously described [37]. The same procedure was repeated on the contralateral limb with the injection of a 1 mL saline control.

In the main PRP effect study, using the previously unused radiocarpal joints, the 0.0625 ng dose of LPS was injected using the same technique; however, the LPS was then followed immediately using the same needle stick with 2 mL of PRP contained within a separate syringe. The same procedure was repeated on the contralateral limb with LPS and 2 mL of saline control.

### 2.4. Synovial Fluid Analysis

Synoviocentesis was performed to obtain approximately 3 mL of SF where possible at PIH 0, 8, 24, 72 and 168. Total protein and white blood cell counts were performed on all samples whilst samples from the main study also underwent biomarker (TNF-α, PGE_2_, MMP and GAG) analysis as previously described [37].

#### Platelet-Rich Plasma Analysis

Levels of TGF-β1 and PDGF-BB were quantified in equine platelet-rich plasma (PRP) using commercially available human ELISA kits (Quantikine™ Human TGF-β1 and PDGF-BB Immunoassay; R&D Systems^®^, Minneapolis, MN, USA; Cat. Nos. [SB100C] and [SBB00], respectively), previously used in horses [46,47,48,49]. Prior to assaying, TGF-β1 samples were activated using a commercial Sample Activation Kit (R&D Systems^®^, Minneapolis, MN, USA; Cat. No. DY010); in summary, 40 μL of PRP was mixed with 10 μL of 12 N hydrochloric acid in a polypropylene tube, homogenised and incubated at room temperature for 10 min. Thereafter, 10 μL of 10 N sodium hydroxide was added, followed by a further 10 min incubation period. Following activation, sample pH was verified at 7.2–7.6. Kits were run as per manufacturers guidelines, with the exception of the final step of the PDGF-BB assay, where the solution was aspirated and dispensed five times upon addition of the stop solution to ensure homogeneity. TGF-β1 and PDGF-BB samples were analysed at a final dilution of 1:60 and 1:20, respectively. Optical density was measured within 30 min at a wavelength of 450 nm and 570 nm using a microplate reader (Absorbance 96; Byonoy GmbH, Hamburg, Germany).

### 2.5. Clinical Evaluations

#### 2.5.1. Welfare Monitoring

A composite welfare scoring (CWS) system was utilised at all of the 12 timepoints (PIH 0, 2, 4, 6, 8, 24, 48, 72, 96, 120, 144, 168) as previously reported [32,37].

#### 2.5.2. Clinical Measurements

Carpal joint effusion score (JES), passive carpal flexion score (FS), and carpal circumference (CC) were performed at each of the 12 timepoints by the same blinded assessor (MD) as previously described [37].

### 2.6. Lameness Evaluation

Subjective lameness grading (SLG) and objective lameness evaluation were performed at the trot as previously been described [37].

#### 2.6.1. Thermography

An InfraRed Variocam HD (InfraTec GmbH, Dresden, Germany) was used to acquire still thermographic images as previously described [37] at PIH 0, 2, 4, 6, 8, 24, 48, 72, 96, 120 and 168.

#### 2.6.2. Statistical Analysis

A sample size calculation was used to estimate the required number of horses based on previous work by this research group when an 80% power and 0.05 level of significance were assumed. The calculation estimated between 7 and 8.1 animals per group were necessary. As a result, 9 horses were used to allow for attrition.

A linear mixed model was performed with horse treated as a random effect for each model; fixed effects were set as time, treatment, and interaction. Model residuals were checked to satisfy assumptions of normality and randomness. If the assumptions were violated, then appropriate data transformations were performed. Residuals being normally distributed were assessed holistically using appropriate numerical descriptive statistics (mean, median and skewness), graphical descriptive statistics (QQ plot, histogram) and statistical tests (Shapiro–Wilk test). Residuals being random were assessed using a scatterplot of fitted values against residuals, along with a statistical test (Breusch–Pagan test). Tukey’s Honest Significant Difference (HSD) was used for pairwise comparisons. Raw TNF-α data was extremely over-dispersed, with no appropriate data transformation possible. Hence, a between-subjects factor made of all possible combinations to treatment with timepoint was made, applying a Kruskal–Wallis test followed by post hoc analysis controlled for multiple comparisons using Dunn’s test. Due to the ordinal/binary nature of FS, JES and SLG data, the relationship was assessed graphically—no formal statistical analysis was performed. All statistical tests are interpreted using a 5% level of significance (*p* < 0.05). Statistical analysis was performed using R statistical programming language in RStudio (version 2026.07.1+147) [50]. All data was included in the analysis unless specifically stated.

## 3. Results

### 3.1. Preliminary Study

**Figure 3:**
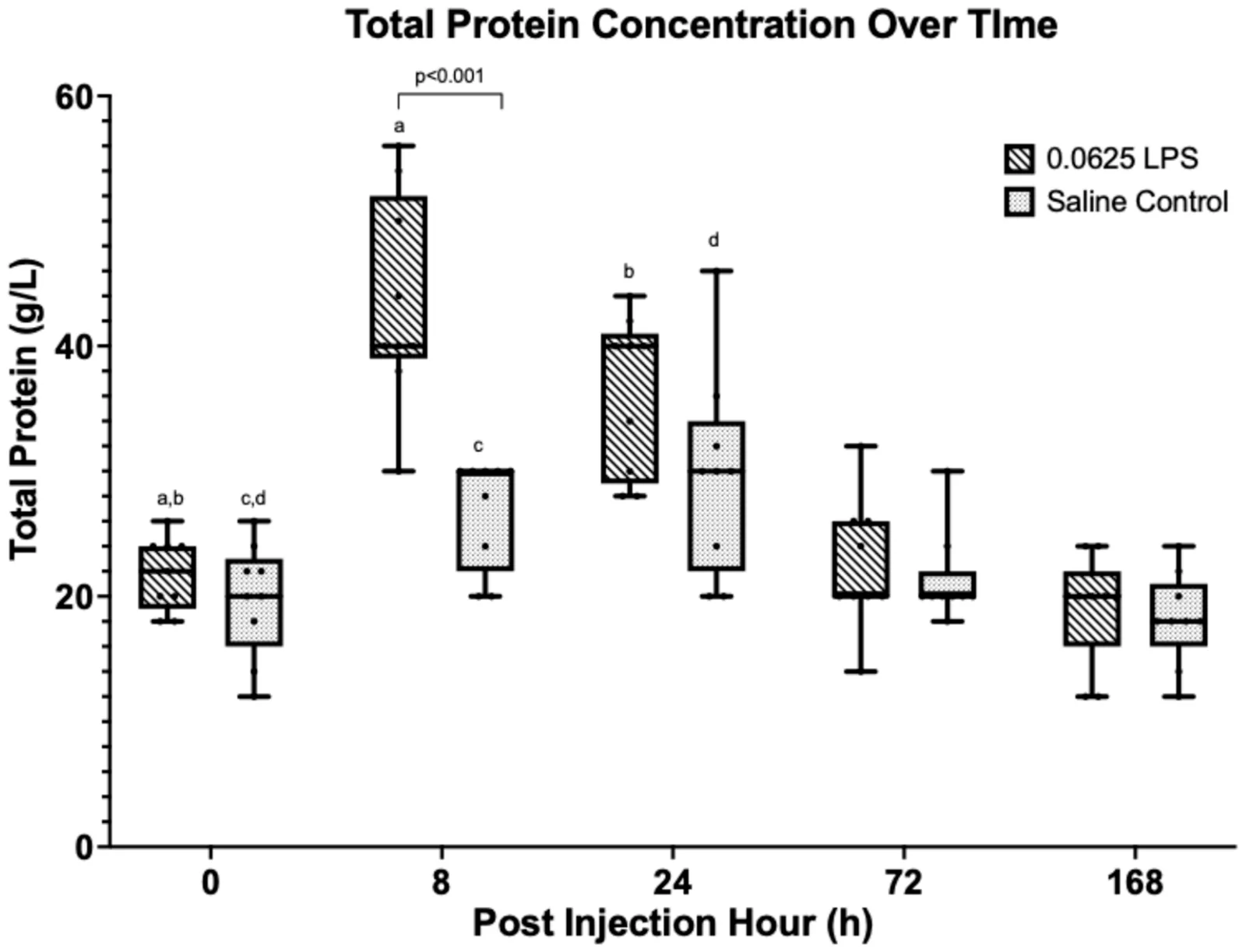
Synovial fluid TP over time between treatment groups. Statistical significance between treatment groups exists, as denoted by their respective *p*-values. Lower-case letters denote statistically significant differences from baseline, a–d (*p* < 0.001).

**Figure 4:**
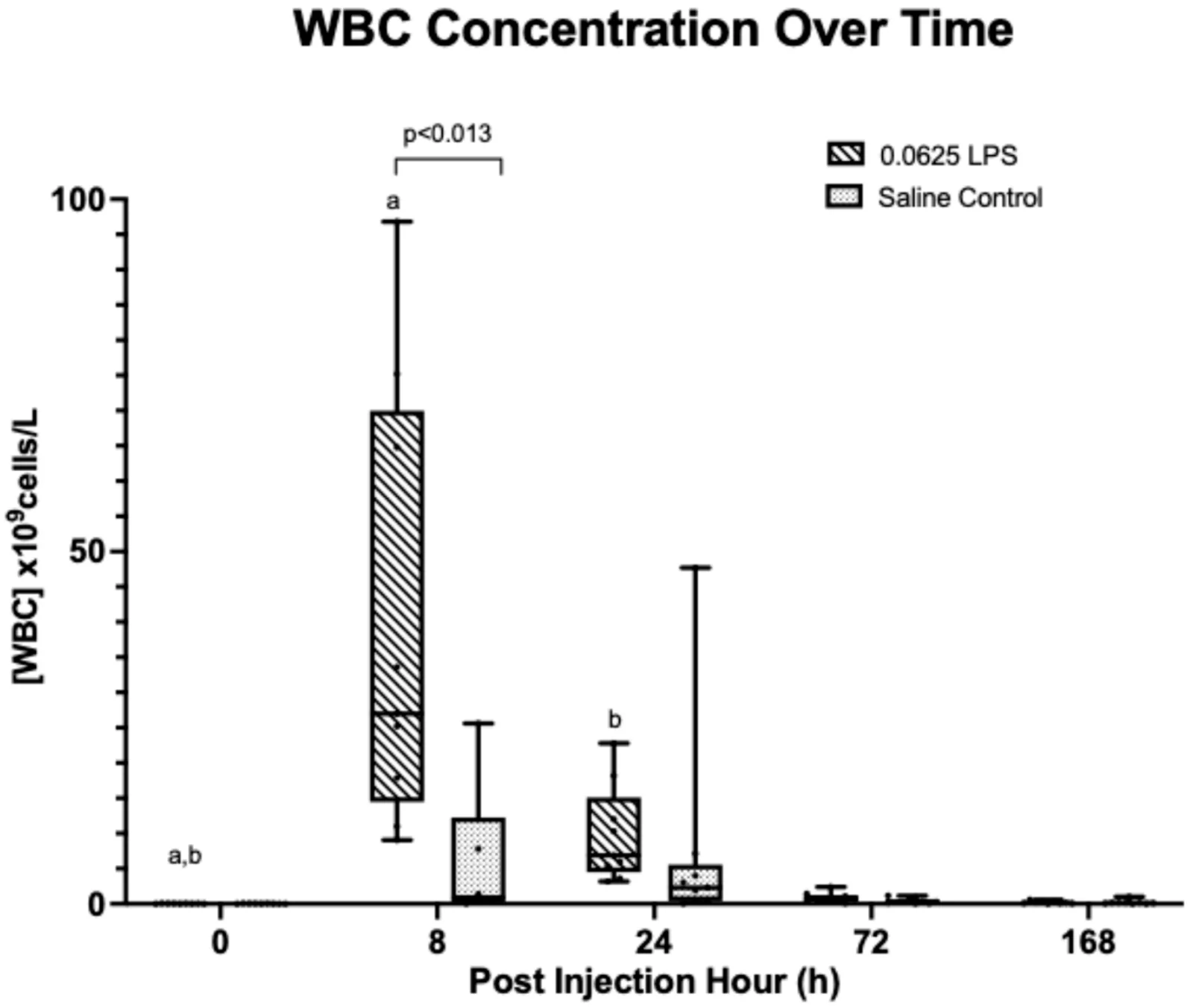
Synovial fluid WBC concentrations over time between treatment groups. Statistical significance between treatment groups exists, as denoted by their respective *p*-values. Lower-case letters denote statistically significant differences from baseline, a–b (*p* < 0.001).

A statistically significant increase in synovial total protein (TP) [Figure 3] and white blood cell (WBC) [Figure 4] concentrations from baseline at PIH 8 (*p* < 0.001) and 24 (*p* < 0.001) were detected, with a return to baseline values by PIH 72 in the 0.0625 ng group. A statistically significant difference between the 0.0625 ng LPS and control group was detected at PIH 8 (TP (*p* < 0.001), WBC (*p* = 0.013)). There were no significant increases in HR, RR or Rectal Temperature across any timepoint.

### 3.2. Platelet-Rich Plasma Effect Study

#### 3.2.1. PRP Composition

The cellular composition of the donor blood and the PRP produced, both before and after freezing, are shown in Table 1. The final PRP product has been classified as per-published human classification systems and summarised in Table 2 based on automated, haematological analysis.

#### 3.2.2. Welfare Monitoring

There were only marginal increases in CWS across timepoints which had returned to baseline values by PIH 24 in all cases. No formal statistical analysis was performed.

#### 3.2.3. Clinical Measurements

No statistically significant differences in CC over time between treatments were detected. However, a treatment effect was present as a main effect only (*p* = 0.003). A statistically significant increase in CC from baseline was detected at PIH 6, with timepoint as a main effect only. Formal statistical analysis on JES, SLG or FS was not performed due to the ordinal nature of the data. However, upon graphical assessment, there were higher flexion and joint effusion scores recorded in the PRP group with peaks at PIH 4 and 6, respectively. Subjective lameness grades were higher at PIH 2, 4, 6 and 8 in the PRP group when compared with the control. There were no significant increases in HR, RR or Rectal Temperature across any timepoints.

#### 3.2.4. Synovial Cytology

**Figure 5:**
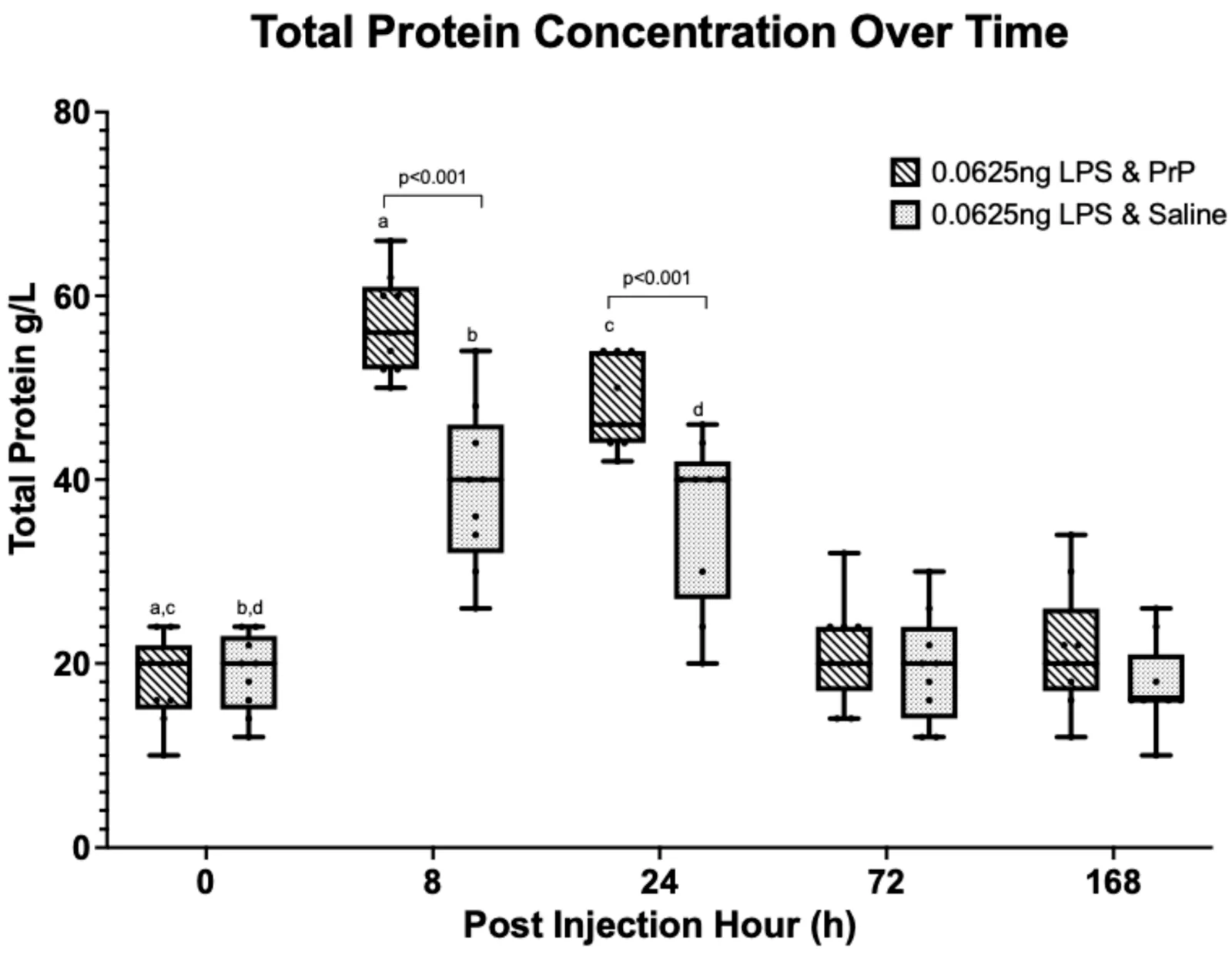
Synovial fluid TP over time between PRP and control groups. Statistical significance between treatment groups exists, as denoted by their respective *p*-values. Lower-case letters denote statistically significant differences from baseline, a–d (*p* < 0.001).

**Figure 6:**
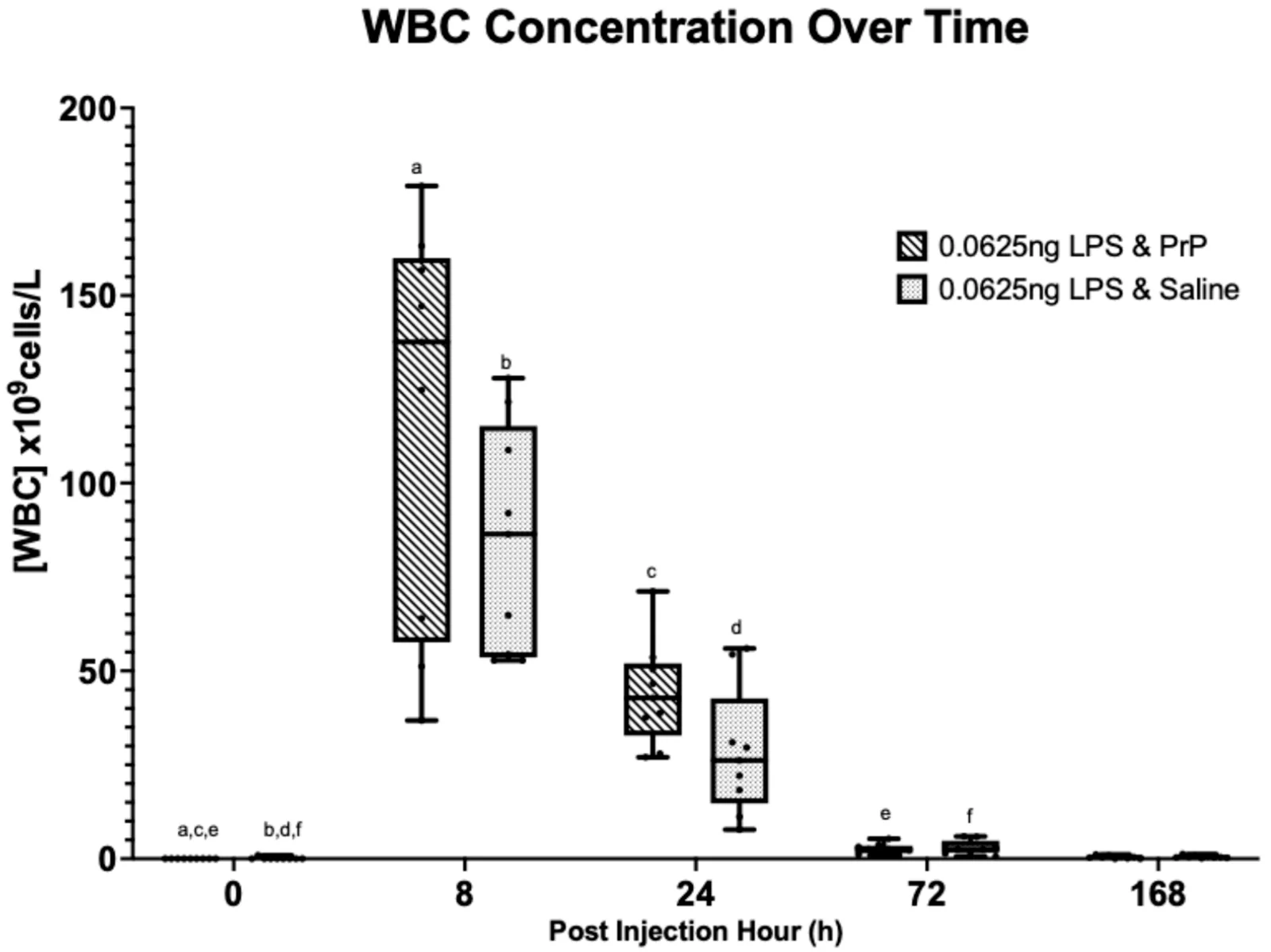
Synovial fluid WBC over time between treatment groups. Statistical significance between treatment groups exists, as denoted by their respective *p*-values. Lower-case letters denote statistically significant differences from baseline, a–f (*p* < 0.001).

The intra-articular administration of both the LPS PRP treatment and the LPS saline control caused significant increases (*p* < 0.001) in TP and WBC concentrations in synovial fluid post-injection, both peaking at PIH 8. Interestingly, the PRP treatment was unable to reduce either of these markers, and in fact, the opposite was found. There was a statistically significant increase (*p* < 0.001) in the synovial TP and WBC concentrations from baseline at PIH 8 and 24 in the PRP group with a return to baseline values by PIH 72. A statistically significant difference in TP between the PRP and control groups existed at both timepoints (*p* < 0.001) [Figure 5]. No statistically significant increase in the synovial white blood cell concentration from control was detected at any timepoint despite significant increases from baseline at PIH 8, 24 and 72 (*p* < 0.001) [Figure 6].

#### 3.2.5. Synovial Biomarkers

**Figure 7:**
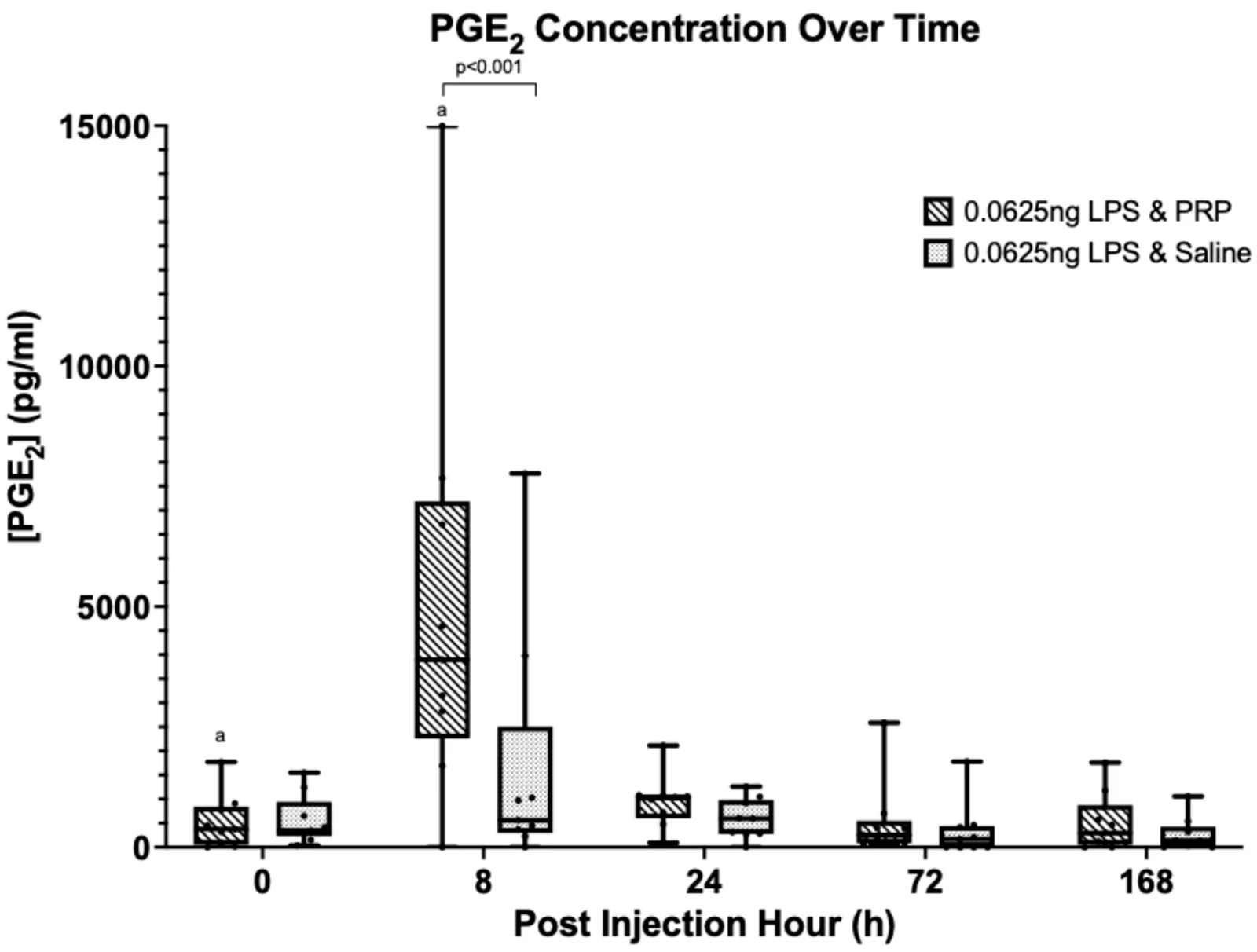
Synovial fluid PGE_2_ over time between treatment groups. Statistical significance between treatment groups exists, as denoted by their respective *p*-values. Lower-case letters denote statistically significant differences from baseline, a (*p* < 0.001).

**Figure 8:**
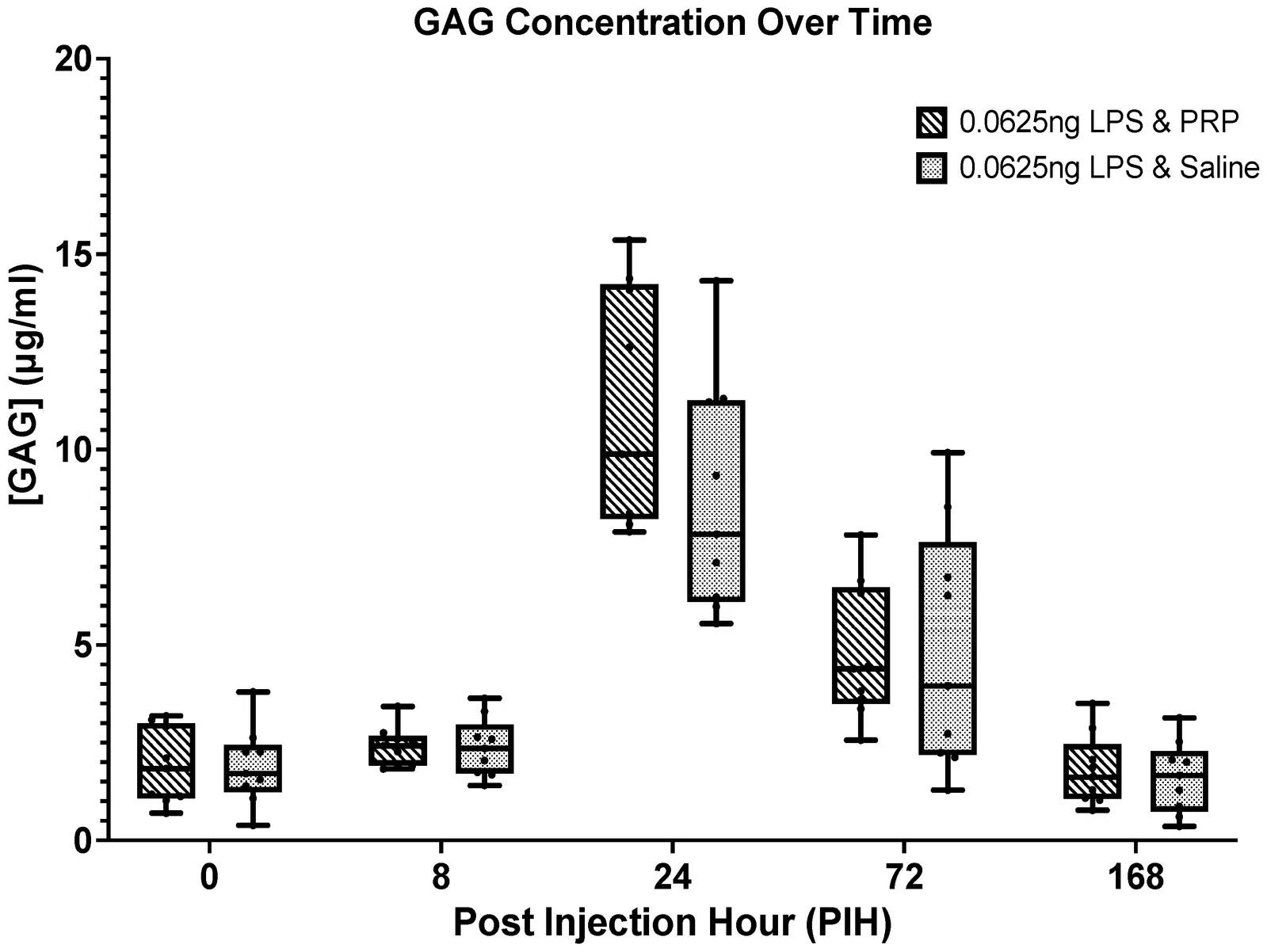
Synovial fluid GAG concentration over time between treatment groups. A statistically significant increase in the synovial GAG concentration was detected at PIH 24 (*p* < 0.001) and 72 (*p* < 0.001), with timepoint as a main effect.

There was a statistically significant increase in the synovial PGE_2_ from baseline at PIH 8 in the PRP group (*p* < 0.001) and between treatments at PIH 8 (*p* < 0.001) [Figure 7]. This increase had returned to baseline values by PIH 24. A statistically significant increase in synovial MMP activity was detected in the PRP treated group with treatment as a main effect only (*p* = 0.035) when compared with the control. A statistically significant increase in the synovial GAG concentration was detected at PIH 24 (*p* < 0.001) and 72 (*p* < 0.001), with timepoint as a main effect [Figure 8]. A statistically significant increase from baseline TNF-α was detected at PIH 8 in the PRP group (*p* < 0.001) but not the control (*p* = 0.114). There was no difference between peak values at PIH 8 (*p* = 0.408).

#### 3.2.6. Objective Gait and Thermographic Analysis

No statistically significant differences in lameness from baseline values were detected at any timepoint. A statistically significant increase in absolute temperature difference was detected when timepoint (*p* = 0.002) and treatment (*p* = 0.02) were considered as main effects only.

## 4. Discussion

In this study, we found that the 0.0625 ng dose of LPS is sufficient to induce a measurable degree of inflammation based on consistently increased TP and WBC. Using this LPS model of joint inflammation, we did not detect any significant anti-inflammatory effects of PRP treatment.

Prostaglandin E_2_ was transiently increased in our study at PIH 8, which is similar to Garbin et al., who reported an increase at PIH 6 when using a similar allogenic PRP preparation in healthy equine joints [23]. Increases in PGE_2_ after injecting PRP in healthy joints have also been reported by others, ranging from PIH 3 to 48 [57]. The PGE_2_ concentration increased to much higher levels in the PRP treatment group compared to the control plus LPS; this would suggest the increase in PGE_2_ truly resulted from the PRP treatment. Whether that was due to increased PGE_2_ within the PRP or increased production of synovial PGE_2_ induced by the PRP has yet to be determined. However, it has been shown that activated platelets release PGE_2_ that promote IL-10 and suppress TNF-α production in monocytes/macrophages [58]. This initial PGE_2_ increase may simply reflect the acute inflammatory response phase. Further analysis of PRP biomarkers would be advantageous in ascertaining the origin of the PGE_2_ in this instance and in identifying other potentially beneficial biomarkers.

In this study, whilst there was a significant increase in TNF-α compared to baseline values in response to LPS, no significant effect of PRP treatment on TNF-α was found. This is consistent with the findings of Moraes et al. (2015) [57]. Increased PRP leukocyte concentrations have been associated with increased IL-1 and TNF-α in other studies [59], but the leukocyte concentration in the PRP used in this study was low, making the PRP itself a less likely explanation for the lack of reduction [60,61]. A meta-analysis looking at the effects of PRP on the inflammatory processes within joints reported that PRP tends to result in reduced TNF-α. However, this was based on very low-quality evidence with poor quality study design [20].

The increase in MMP activity when treatment was considered as a main effect is interesting as PRP has previously been shown to contain varying levels of MMPs [62,63,64]. Given the potential to accelerate cartilage catabolism and the effects that preparation variables such as freeze–thaw have on PRP MMP composition, this warrants further investigation [64]. A more recent publication by Fukuda et al. concluded that platelet lysate did not demonstrate any anti-inflammatory properties when studied in a monoiodoacetic acid (MIA) model [65]. On the basis of this finding and recent reports, there is mounting evidence to suggest that PRP does not exert any anti-inflammatory effects and that it, in fact, might be pro-inflammatory.

The results of the preliminary study confirmed that an LPS dose of 0.0625 ng can induce measurable inflammation within equine middle carpal joints with peak increases in TP and WBC at PIH 8 and a return to baseline values by PIH 72. The peak increases in TP and WBC in this study are comparable to those of an earlier study [37] by this research group comparing 0.25 ng and 0.125 ng doses of LPS, suggesting that a 0.0625 ng dose produces a comparable inflammatory response in terms of TP and WBC. The 0.0625 ng group returned to baseline values by PIH 72, as did the 0.125 ng dose in the earlier study [37]. These findings support the authors previous conclusion that a further reduction in LPS may be possible. The preliminary study was valuable in confirming that the 0.0625 ng dose of LPS was sufficient in inducing an adequate cytological inflammatory response in the chosen cohort of horses comparable to that of the 0.125 ng dose in a previous study by this research group [37], given the known potential for both batch and interhorse variation [33]. The 0.0625 ng dose of LPS did not cause any significant lameness and therefore, from a welfare and refinement perspective, is preferable over earlier used higher dosages causing similar inflammation. Nevertheless, we feel that using a preliminary study to determine LPS dosage effect in the actual experimental cohort of horses is beneficial when testing the effect of anti-inflammatory treatments, especially since variation in response is a common limitation of the LPS models in large animals.

The increase in TP in the PRP-injected joints at the peak timepoint PIH 8 compared to control should be interpreted cautiously as the PRP that was injected contained protein (58.4 g/L). It is, therefore, likely that the increase in protein (mean ± SD 56.89 ± 5.40 g/L) above the control value (mean ± SD 39.11 ± 8.78 g/L) is, at least in part, due to this exogenous protein. However, platelet-rich preparations have been shown to increase hyaluronic acid secretion by synovial fibroblasts [66,67] with PDGF-BB and TGF-β1 contributing to the upregulation of hyaluronan synthase isoforms 1 and 2 [68,69]. This would suggest that PRP is capable of inducing synovial biosynthetic processes, which in turn may result in the synthesis of other bioactive components, contributing to TP increases rather than being exclusively from an inflammatory response or exogenous source. However, this was not determined in this study. There was no significant increase in WBC compared with the control values suggesting that any leukocytosis caused was the result of the LPS, implying that PRP does not cause any additional increases in WBC. The increases in WBC with the LPS control, whilst not significantly different, are still at a magnitude much greater than what would typically be seen with clinical diseases such as equine degenerative joint disease where values of 5–10 × 10^9^ cells/L are reported [70], with values as low as 0.034–0.141 × 10^9^ cells/L reported [71]. This represents a limitation of this model and the main reason why we are trying to reduce the LPS dose as much as possible to lower the level of inflammation.

Neither the 0.0625 ng dose of LPS nor the PRP induced any significant changes in objective lameness measures from baseline. These findings must be considered with caution given the bilateral injection of LPS in that study. However, the experimental benefit of a within-animal controlled design far outweighs this limitation. In addition, the aim of our LPS study design is to cause minimal but reliable inflammation whilst having minimal negative welfare effects (lameness will be an indicator of joint pain). When assessing the subjective lameness grades, the PRP group were subjectively lamer across PIH 2, 4, 6 and 8 compared to the LPS plus saline control group. While there were no significant changes from baseline using the objective measurements there was a marked increase in variation across PIH 2, 4 and 6, which may have become significant with a larger study population. These findings are not definitive but suggest that PRP may induce a transient, low-grade lameness in some horses compared with a saline control, which may correspond with the increased PGE_2_ at PIH 8. This finding would be consistent with the “flares” that are reported following intra-articular injection of PRP in a clinical setting, which is reported to occur in 2–10% of horses [72].

The statistically significant difference in absolute temperature difference when timepoint was considered as a main effect only is supportive of the previously reported finding that arthrocentesis can be detected using thermography [73]. Previous work by this group [37] demonstrated that thermography was able to detect differences in the duration of synovitis caused by two different doses of LPS.

Platelet-rich plasma is a blood-derived product in which RBCs are removed and the platelet fraction concentrated above that of baseline whole blood values [8,9]. The production of PRP can utilise an array of different commercial kits and lab-based centrifugation techniques. However, the processes used are not standardised, nor is the definition of PRP. The technique chosen in this study was adapted from Garbin et al. (2022) [23], who studied the use of allogenic freeze-dried PRP in healthy equine joints. The authors felt this was valuable as it offered some continuity in the protocol used, and the product that was produced was proven to be safe in healthy joints. Therefore, this negates the need for a prior safety study. Furthermore, the use of an allogenic preparation offered significant advantages in standardisation of the study design as it is well documented that intrinsic factors (e.g., breed, age, gender) affect PRP quality with greater platelets in females and increased PDGF-BB in horses less than 5 years of age [24]. A young (4-year-old) Thoroughbred mare was used as a donor to optimise PRP quality. The use of allogeneic preparations, while having previously been shown to be safe [23,74], could introduce the potential for immune-mediated reactions, which may influence the host inflammatory response. Further investigation of this was beyond the scope of the study. The protocol used in this study differed slightly from that used by Garbin et al., in that freeze-drying was not performed and the produced PRP was simply frozen at −80 °C. The authors felt this was more widely reproducible and clinically applicable.

The final PRP composition [Table 1] using the above-mentioned protocol resulted in what we believe to be “good”-quality PRP based on the available literature to date. Normal equine whole blood has a platelet concentration ranging from 100-to-300 × 10^3^ platelets/μL [19], and it has been reported in humans that PRP with a platelet count of 1 × 10^6^ platelets/μL is supportive of healing [75]. The optimal platelet count in PRP is suggested to lie between 3 and 9 times that of whole blood [19,76]. However, the ideal multiplication factor has not yet been determined [21,77]. Some authors have suggested that intermediate platelet concentration (2–6 × whole blood) should be favoured [78,79], with marked increases in platelet count potentially detrimental to growth factor activity through a proposed negative feedback mechanism [19]. The protocol used in this study generated PRP with a mean platelet count of 954 × 10^9^ cells/L compared with a mean blood platelet count of 158 × 10^9^ cells/L, resulting in a platelet multiplication factor of 6.04. Platelet concentrations were measured using an automated analyser (Advia 2120, Siemens Healthcare Diagnostics Inc., New York, NY, USA) which has previously been validated for counting of equine platelets [45]. TGF-ß1 concentrations were comparable (although higher) to those reported by Fukuda et al. [38], and significantly greater than McClain et al. [80], whereas the inverse was true for PDGF-BB. TGF-ß1 and PDGF-BB values in this study were considerable higher than those reported after freeze–thaw by Textor et al. [81] by a factor of ~2.5 and ~4, respectively, which likely reflects protocol or horse variation.

Red blood cell contamination in PRP should be limited due to the marked pro-inflammatory action induced by the release of macrophage migration inhibitory factor caused by eryptosis [82]. Furthermore, damaged RBCs release haemoglobin and subsequent degradation factors that are cytotoxic [82]. The administration of RBCs intra-articularly resulted in the release of catabolic mediators (IFN-γ and IL-1) and subsequent synoviocyte death [83]. Red blood cell contamination was minimal at 0.01 × 10^12^ cells/L a reduction of 953 times compared to whole blood using this protocol.

The presence of leukocytes (particularly neutrophils) in PRP is a much-debated topic, with some reports showing that the inclusion of leukocytes is associated with increased catabolic gene expression and cytokines release [56,84]. To minimise pro-inflammatory cytokine accumulation at detrimental levels, leukocyte ranges of 0.1–3 × 10^3^ cells/μL have been reported [56]. One study showed neutrophils were directly correlated to increased MMP-9, a proteinase associated with inferior wound healing [84]. Braun et al. (2014) concluded that leukocyte-poor PRP was favourable for intra-articular use due to greater anti-inflammatory cytokine activation compared to pro-inflammatory equivalents and significantly greater synoviocyte death in the leukocyte-rich PRP group [83]. Leukocyte concentration was reduced from 11.45 × 10^9^ cells/L in WB to 1 × 10^9^ cells/L in PRP with a 20.3-times reduction in neutrophils from whole blood to 0.4 × 10^9^ cells/L in our formulation, confirming that this protocol is capable of significantly reducing leukocyte numbers.

Whilst there is a general consensus that neutrophils are detrimental to the effects of PRP, increased monocytes may have anti-inflammatory benefits [19,82]. Monocytes differentiate into macrophages (MΦ), of which there are inflammatory (MΦ1) and anti-inflammatory (MΦ2) phenotypes [85]. This differentiation is influenced by the cellular environment and associated stimuli [85]. MΦ1 are associated with pro-inflammatory cytokine release (IFN-γ) and NO, whereas MΦ2 are closely associated with IL-10, chemokines and other anti-inflammatory products. MΦ2 lineages have high phagocytic potential [85]. PRP studies often fail to report the levels and discuss the role of monocytes despite their potential to contribute to improved healing. Further investigation into the role of monocytes in PRP should be considered. If found to be of significant benefit, this protocol is unlikely to achieve adequate monocyte concentration.

Another key variable in the use of PRP is the means of platelet activation, for which there are many proposed mechanisms. This process results in the secretion of platelet organelle contents and ultimately the exertion of their effects. Activation techniques that are commonly reported include the addition of thrombin, CaCl_2_, a combination of the two [81], freeze–thaw cycling [81,86], addition of thrombin receptor agonist peptide [87], collagen [88], and mechanically by injection [89]. The authors elected to use a single freeze–thaw technique as this more closely represents what is done in clinical practice. However, CaCl_2_ was deemed more effective than bovine-thrombin or single freeze–thaw methods of activation [81]. Conversely, Sonker et al. concluded that a double freeze–thaw method resulted in better activation as it also lysed white blood cells that contain growth factors [86], and this appears to be the case following a single freeze–thaw cycle in this study where both neutrophil and monocyte numbers decreased, yet lymphocyte numbers appear to have increased, maintaining a comparable total WBC count. In retrospect, a double freeze–thaw technique may have been more appropriate and still applicable to clinical practice. It is reported that some clinicians inject PRP without deliberate activation [67,89], suggesting that injection of the final PRP product is sufficient for platelet activation—this is not widely supported by the literature. It was found that there were negligible effects of the shear forces induced by injection through either a 21 g or 25 g hypodermic needle on growth factor release [89], with a subsequent study reporting that the smallest commercially available needle (30 gauge) did not activate platelets [90].

Multiple classification systems exist for describing PRP in humans. However, to date, there are no equivalent, validated classification systems available in veterinary medicine. Establishment of such a system should be a priority to help standardise research in this area. Chahla et al. reported that only 10% of published studies provided sufficient detail on the PRP preparation protocol that would allow repetition and only 16% provided detailed analysis of the end PRP product [91]. Therefore, the authors feel it is paramount that detailed information be included in all future publications regarding orthobiologics to allow direct comparisons to be made and further work to be performed to enhance our understanding of these products. Hence, the data included here allows classification using these already proposed systems where possible.

The lack of a clear definition of what is PRP means the results of this study are only applicable to this specific product, and comparison across other studies is likely to be unreliable. The authors have made a concerted effort to be open and transparent regarding the PRP protocol and composition. However, further, more detailed analysis of the PRP product used in this study would be advantageous.

A limited subset of synovial biomarkers was analysed in this study, and further expansion of this would be advantageous, as would the inclusion of a validated equine synovial multiplex assay. The analysis was restricted to five timepoints, which leaves significant time periods open to speculation. The study results are only applicable to a Thoroughbred population as a result of the apparent breed specific lameness response to LPS.

## 5. Conclusions

In conclusion, the PRP used in this study did not display any measurable anti-inflammatory properties during the early inflammatory phase when assessed using the described analytical measures. In fact, it may be suggested that PRP is pro-inflammatory during this phase. The biomarker panel analysed was limited, and further work should be undertaken to identify the optimal biomarker array and to enhance the clinical relevance of the LPS model at this lower 0.0625 ng dose. The PRP used in this study does appear to induce a transient increase in PGE_2_, which may be reflective of the transient synovial “flare” that is intermittently reported clinically. Further work is required to ascertain the relationship between PRP and this transient increase in PGE_2_ and the clinical relevance of this relationship.

## Figures and Tables

**Table 1:** Summary of blood and PRP parameters with the associated multiplication or reduction factor indicated. (HCT, Haematocrit; RBC, Red Blood Cells; PLT, Platelets; WBC, White Blood Cells; Neut, Neutrophils; Mono, Monocytes; Lymp, Lymphocytes; TP, Total Protein; Alb, Albumin; Glob, Globulin).

	HCT (%)	RBC (×10^12^/L)	PLT (×10^9^/L)	WBC (×10^9^/L)	NEUT (×10^9^/L)	MONO (×10^9^/L)	LYMP (×10^9^/L)	TP (g/L)	Alb (g/L)	Glob (g/L)	PDGF-BB (pg/mL)	TGF-ß1 (pg/mL)
**Blood**	41.00	9.53	158.00	11.45	8.13 (71%)	0.46 (4%)	2.63 (23%)					
**PRP**	0.00	0.01	954.00	1.00	0.4 (40.4%)	0.03 (2.6%)	0.49 (48.8%)					
**Reduction/Multiplication Factor**	41.00	953.00	6.04	11.45	20.24	15.33	5.37					
**PRP Post Freeze–Thaw**	0.00	0.04	809.75	0.98	0.15 (15.4%)	0.01 (1.3%)	0.78 (79.9%)	58.40	32.00	26.40	7889.76	27,369.96

**Table 2:** Classification of the final PRP product using the classification systems reported in the literature.

System	Classification [Based on Mean Values]	Reference
**DEPA**	CCA	[51]
**PAW**	P3-FT-Bβ (FT = Freeze–Thaw)	[52]
**Code**	190010	[53]
**Sports Medicine**	Type 3-A	[54]
**Dohan**	Leukocyte Poor PRP (LP-PRP)	[55]
**PLRA**	P(954)L(+)(40.4%)R(−)A(+)	[56]

## Data Availability

The data is available upon reasonable request from the corresponding author due to ethical restrictions.
